# Ultrafast formation of air-processable and high-quality polymer films on an aqueous substrate

**DOI:** 10.1038/ncomms12374

**Published:** 2016-08-10

**Authors:** Jonghyeon Noh, Seonju Jeong, Jung-Yong Lee

**Affiliations:** 1 Graduate School of Energy, Environment, Water, and Sustainability (EEWS), Graphene Research Center, Korea Advanced Institute of Science and Technology (KAIST), Daejeon 305-701, Republic of Korea

## Abstract

Polymer solar cells are attracting attention as next-generation energy sources. Scalable deposition techniques of high-quality organic films should be guaranteed to realize highly efficient polymer solar cells in large areas for commercial viability. Herein, we introduce an ultrafast, scalable, and versatile process for forming high-quality organic films on an aqueous substrate by utilizing the spontaneous spreading phenomenon. This approach provides easy control over the thickness of the films by tuning the spreading conditions, and the films can be transferred to a variety of secondary substrates. Moreover, the controlled Marangoni flow and ultrafast removal of solvent during the process cause the films to have a uniform, high-quality nanomorphology with finely separated phase domains. Polymer solar cells were fabricated from a mixture of polymer and fullerene derivatives on an aqueous substrate by using the proposed technique, and the device exhibited an excellent power conversion efficiency of 8.44 %. Furthermore, a roll-to-roll production system was proposed as an air-processable and scalable commercial process for fabricating organic devices.

## Abstract (web summary)

There is a continual effort to improve the power conversion efficiency of polymer solar cells and here, Lee and others have produced a device with 8.44% efficiency. This was achieved by using a spontaneous spreading phenomenon to form uniform, high quality films with finely separated phase domains.

## Graphical abstract

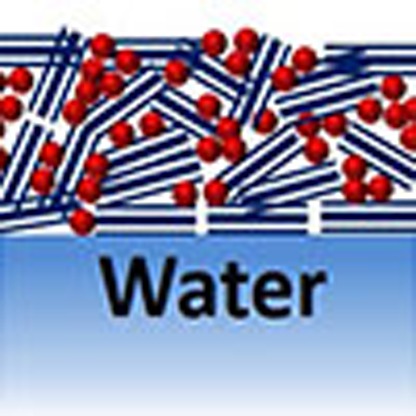

Control of the nanomorphology in polymer solar cells (PSCs) is a key factor for maximizing the module power conversion efficiency (PCE) and enhancing the electrical properties related to exciton dissociation at the interfaces between donors and acceptors^1^,^2^,^3^, diffusion of charge carriers^4^,^5^, and charge collection at each electrode^6^,^7^,^8^. The nanomorphology of the blended film can be engineered via thermal and solvent annealing^9^,^10^ or the use of processing additives and so on, to achieve selective solubility^11^. Effective manipulations may favorably influence the crystallization and orientation of the polymers^12^.

Although spin-coating is the most appropriate laboratory-scale film formation process, scale-up of this process does not ensure uniformity, even within a device. This makes the spin-coating process incompatible with roll-to-roll (R2R) production under ambient conditions, where the latter is desirable for successful commercialization.

Furthermore, air processability of PSCs is a critical issue given that an inert environment is currently required for the entire process, unless the devices are stable under ambient conditions. Although poly(3-hexylthiophene) (P3HT)-based devices exhibit good air processability due to reversible degradation by oxygen, highly efficient polymers containing the benzodithiophene (BDT) group, such as poly[[4,8-bis[(2-ethylhexyl)oxy]benzo[1,2-b:4,5-b']dithiophene-2,6-diyl][3-fluoro-2-[(2-ethylhexyl) carbonyl]thieno[3,4-b]thiophenediyl]] (PTB7) and poly[4,8-bis(5-(2-ethylhexyl)thiophen-2-yl)benzo[1,2-b;4,5-b']dithiophene-2,6-diyl-alt-(4-(2-ethylhexyl)-3-fluorothieno[3,4-b]thiophene-)-2-carboxylate-2-6-diyl)] (PTB7-Th), tend to undergo significant degradation on exposure to oxygen and light^13^,^14^,^15^,^16^.

Here we propose a new approach to form high-quality organic films on an aqueous (water based) substrate under ambient conditions. A drop of organic material blended into a solvent, when dropped onto an aqueous surface, spreads spontaneously and rapidly by the Marangoni effect^17^, leading to the rapid and uniform formation of an organic layer that can be functionalized on various substrates once transferred. Interestingly, the nanomorphology of blended films can be efficiently managed in seconds during the process of spreading on the aqueous substrate. We verify that the ultrafast solvent removal by the spontaneous spreading process can impede the infiltration of oxygen into the film via the remaining solvent effectively.

The utility of the films in functional devices is demonstrated by applying the new film formation technique to the fabrication of highly efficient PSCs. PTB7-based PSCs are successfully fabricated by exploiting the high-quality nanomorphology within the bulk heterojunction (BHJ) films. Last, we demonstrate the potential of our method as a scalable process by transferring the spontaneously spreading (SS) film from water surface to a large plastic substrate using a custom-made R2R process.

## Results

### Spontaneously spreading process of polymer solutions

**Figure 1:**
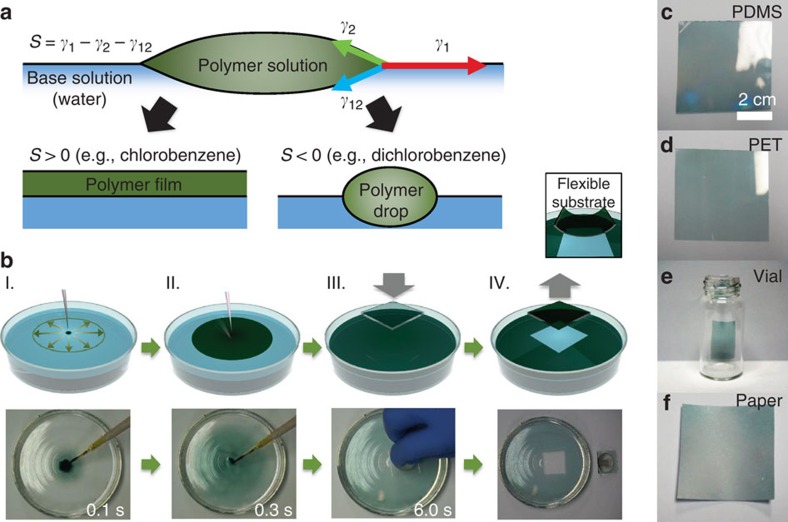
Principle and formation process of spontaneous spreading. (**a**) Spreading of polymer solution dropped onto the base solution is determined by the spreading coefficient, *S*. Positive *S* results in a uniform polymer film; otherwise, polymer drops aggregate; (**b**) Schematic illustrations of formation of an SS BHJ film on water and transfer to target substrates. Actual processing images are shown. (**c**–**f**) Transferred SS-PTB7:PC_71_BM films on various substrates: PDMS (**c**), PET (**d**), copy paper (**e**) and a curved surface of a vial (**f**).

When chemical agents with low surface energy (for example, hydrocarbon solvents or detergents) are dropped into high-surface-energy media (for example, water), local surface tension gradients occur on the boundary between the surrounding materials, leading to surficial flow toward regions of higher surface tension. This spontaneous spreading is called Marangoni flow^17^,^18^. The spreading coefficient (*S*) dictates the speed of the spreading flow and is defined by the surface tensions at the three-phase contact line between a liquid droplet and liquid substrates^19^, where *S*=*γ*_1_–*γ*_2_–*γ*_12_ (*γ*_1_ and *γ*_2_ are the surface tensions of the base and polymer solutions, respectively, and *γ*_12_ is the interfacial surface tension of the two solutions). Figure 1a illustrates the different behaviours of the polymer solution depending on *S* when a drop of polymer solution is dropped on the base solution. The flow of the solution is determined by the net force direction of the surface tensions. If *S* is positive, that is, the surface tension of the base solution, *γ*_1_ (red arrow), is larger than the sum of *γ*_2_ (green arrow) and *γ*_12_ (blue arrow), the polymer solution will spread over the aqueous substrate surface and form a uniform polymer film. Otherwise, the polymer solution will ball on the aqueous substrate without dispersion.

The proportion *S*^1/2^ describes the spreading speed of the solution^20^,^21^, and the solution is unlikely to spread if *S* is negative. The spreading coefficients of 1,2-dichlorobenzene (*o*-DCB) and chlorobenzene (CB) are −4.25 and 2.35 dyne cm^−1^, respectively^21^,^22^; thus only CB would exhibit spontaneous spreading flow on water. Dissolution of the solvent in water further accelerates drying and formation of the polymer films on aqueous substrates. Figure 1b illustrates the formation of a SS film on an aqueous substrate (here water is used as the aqueous substrate). (I) The polymer solution is dropped onto the aqueous substrate; (II) the solution quickly spreads (if *S*>0) over the aqueous surface within a few seconds; (III) because the solvents evaporate and dissolve into the liquid simultaneously, the drying process occurs instantaneously to form a thin solid polymer layer (the SS film) on the liquid; (IV) this thin film can readily be transferred to a variety of target substrates, for example, by stamping the substrates on the SS film gently (see Supplementary Movie 1, Supplementary Note 1 and Supplementary Fig. 1).

Because the photoactive films of polymer solar cells have dimensions below the wavelengths of visible light (<∼200 nm), control of the thickness within tens of nanometers is very important for achieving optimized performance^23^. Herein, the SS film formation method is shown to be extremely reliable for controlling the thickness of polymer films over a wide range (tens to hundreds of nanometers). The ultimate thickness of organic films forming on water depends on the volume and concentration of the polymer solution, the size of the water bath, and the number of layers transferred (see Supplementary Note 2 and Supplementary Fig. 2a–d). PTB7:PC_71_BM solution was used for the thickness experiments.

### Uniform and conformal transfer on various substrates

The SS films formed in the water bath exhibited controllable thickness and high uniformity over large areas. The SS-PTB7:PC_71_BM film was deposited in a square PET Petri dish (12 × 12 cm^2^), leading to a thickness of 43.9±4.1 nm (see Supplementary Note 3 with Supplementary Figs 3 and 4) as determined from 16 positions on the substrate.

The SS films were also successfully transferred to various secondary substrates including polydimethylsiloxane (PDMS), polyethylene terephthalate (PET), paper, and the curved surface of a vial by the stamping method (Fig. 1c–f). An interference pattern was still apparent on the polyurethane (PU) substrate with sinusoidal gratings (period: 556 nm) after transfer of the SS films to the substrate (Supplementary Note 4; Supplementary Fig. 5a,b). Scanning electron microscope (SEM) images also confirmed successful formation of the SS films.

### Nanomorphology within BHJ films

The universality of SS film formation was demonstrated by generating the SS films using several types of common polymers. AFM images (Supplementary Note 5 and Supplementary Fig. 6) show that poly(3-hexylthiophene-2,5-diyl) (P3HT), poly[2-methoxy-5-(2-ethylhexyloxy)-1,4-phenylenevinylene] (MEH-PPV), PTB7, and PTB7-Th formed uniform and smooth SS films. The SS phenomenon appeared to be uninfluenced by the presence of PC_71_BM and an additive such as DIO.

**Figure 2:**
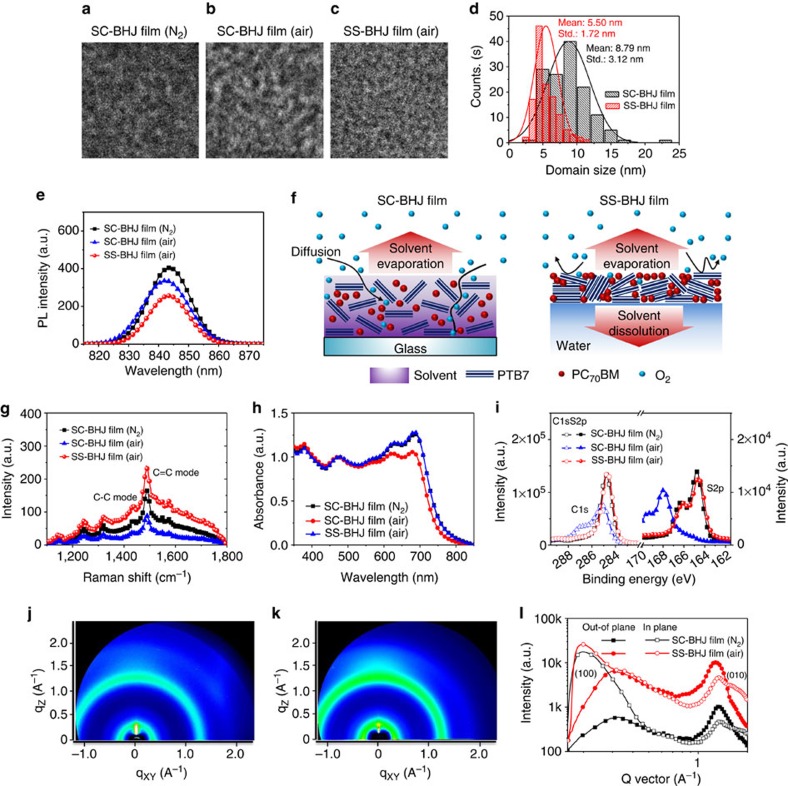
High-quality polymer from spontaneous spreading process. (**a**–**c**) TEM images of N_2_ processed (**a**) air-processed SC-BHJ (**b**) and SS-BHJ (**c**) films measured at the same defocusing distance. (**d**) PTB7 domain size distribution histograms of N_2_-processed SC-BHJ and SS- BHJ films. (**e**) Photoluminescence spectra of both films when excited at 633 nm. (**f**) SC-BHJ and SS-BHJ films immediately after film formation process. (**g**–**i**) Raman spectra (**g**) and ultraviolet–visible absorption spectra (**h**) for SC-BHJ and SS-BHJ films, and S2p and C1s XPS spectra (**i**) of N_2_ and air-processed SC-BHJ, and SS- BHJ films. (**j**–**l**) 2D GIWAXS images of SC-BHJ (**j**) and SS-BHJ films (**k**) and out-of-plane and in plane GIWAXS profiles (**l**).

High-resolution transmission electron microscope analysis of the BHJ films was performed under the same defocused imaging conditions (10 μm) to compare the domain sizes of PTB7 (refs 24, 25). To fabricate top–down transmission electron microscope (TEM) samples, the SS-PTB7:PC_71_BM film was directly transferred to 300 mesh copper TEM grids, while the spin-coated (SC-)PTB7:PC_71_BM films (air and N_2_) were floated on deionized water and transferred. To highlight the PC_71_BM cluster domains (dark regions) and PTB7 crystallite domains (bright regions), the bright regions are coloured red (Supplementary Note 5; Supplementary Fig. 6d–f). The PC_71_BM cluster domains and domains of PTB7 crystallites in the SC-PTB7:PC_71_BM films processed in air and in N_2_ were slightly larger than those of the SS-PTB7:PC_71_BM film (Fig. 2a–c)^26^. Figure 2c illustrates the more uniformly formed nanomorphology of the SS-PTB7:PC_71_BM film with finely dispersed phase separation between the PTB7 and PC_71_BM domains, suggesting efficient spatial interaction between these species during the SS process. The size distribution of the PTB7 domains was determined from at least five samples using ImageJ software (Fig. 2d; see Supplementary Note 6 and Supplementary Fig. 7 for details). The average size of the PTB7 domains in the SS-PTB7:PC_71_BM film was 5.50 nm, while that of the SC-PTB7:PC_71_BM films was 8.79 nm on average; the PTB7 domains in the SS film had a narrower size distribution.

The final morphology of a BHJ film with solvent additives is governed by the dynamics of solvent evaporation and liquid–liquid phase separation^26^,^27^. Immediately on dropping the solution on the water, the solvent (that is, CB) volume decreases due to evaporation and dissolution of the solvent into the water (Fig. 1a); the PTB7 chains start to form aggregated fine structures within a few seconds. This is much faster than the drying of spin-coated BHJ films (a few tens of seconds)^28^.

However, too rapid solvent evaporation and a higher initial concentration of the polymer in the solution at the air/liquid interface may enhance the nucleation rate, leading to a high number of small crystallites^29^,^30^. Therefore, inducing optimized-scale phase separation with appropriately sized crystallites is desirable. To properly control the phase separation, the DIO content in the PTB7:PC_71_BM solution was varied^26^. Because DIO has a higher boiling point (∼170 °C) and higher surface tension (43.25 dynes cm^−1^) than CB, mixing 10 vol% DIO with PTB7:PC_71_BM solution leads to increased surface tension of the droplet, which slows the rate of spreading over the water^31^,^32^. Furthermore, DIO molecules with greater specific gravity (1.84 g cm^−3^) than water are steadily removed from films into water during SS process although DIO is insoluble in water (water solubility at 25 °C: 0.2301, mg l^−1^). This results in sufficient plasticity to allow the aggregated polymer chains to be mobile enough to assume the optimal conformations^31^,^33^. At the same time, the PC_71_BM molecules remain dissolved in DIO, preventing large-scale liquid–liquid phase separation^34^. Subsequently, the PC_71_BM molecules captured in DIO are slowly integrated into the PTB7 crystallites before complete solidification, resulting in larger interface areas between smaller PTB7 and PC_71_BM domains^26^. The larger interfacial areas between domains result in efficient exciton dissociation in the BHJ PSCs, as discussed below^35^.

Figure 2e shows photoluminescence spectra (excited at 633 nm) of the SC- and SS-PTB7:PC_71_BM films on silicon wafers. Significant quenching of the photoluminescence intensity for the SS-PTB7:PC_71_BM film confirms facilitated exciton dissociation in the increased interfacial area between the finely dispersed phase-separated domains, as revealed in the TEM images (Fig. 2c).

The influence of the SS process on the degree of molecular order was probed using resonant Raman spectroscopy (excited at 633 nm) of the SC- and SS-PTB7:PC_71_BM films because these spectra can reveal the intramolecular conformational order of polymer chains (Fig. 2g)^36^,^37^. The wavelength of 633 nm is close to the maximum absorption of PTB7 (Fig. 2h), which allows excitation of even the disordered phase of PTB7 in strong resonance with electronic transitions^37^. The Raman spectra were background corrected and the spectra from three different regions of the samples were averaged. Spectra were also acquired from three samples prepared at different times to exclude sample-to sample variation and measurement error. The Raman peaks of the SS-PTB7:PC_71_BM film at ∼1435, cm^−1^ (C–C intra-ring stretch mode) and at ∼1,490 cm^−1^ (symmetric C=C stretch mode) were almost identical to those of the SC-films. The ratio of the intensity of the peaks of the C–C and C=C modes (*I*_C–C_*/I*_C=C_ of the SS film) increased from 0.46 to 0.49, and the full width at half maximum (FWHM) of the C=C mode became narrower, reflecting the enhanced degree of molecular order of the PTB7 chains in the SS film.

Grazing-incidence wide-angle X-ray scattering (GIWAXS) was also used to verify enhanced crystallization and orientation of the molecules during the spontaneous spreading process. The intensity of the (010) scattering peak at 1.6 Å^−1^ for the SS-PTB7:PC_71_BM film increased markedly compared with that of the SC-PTB7:PC_71_BM film (Fig. 2j–l), indicating enhanced face-on orientation of the PTB7 chains in relation to the substrate in the SS-PTB7:PC_71_BM film processed in air. The enhanced face-on orientation of the SS-PTB7 film is most likely due to the interplay of Van der Waals interaction from alkyl side chains and the attractive interactions occurring between the less hydrophobic backbone of PTB7 and the water surface^38^,^39^. The intensity ratio (*I*_*out*_*/I*_*in*_) of the out-of-plane to in-plane PTB7 lamellar peaks (100) at around 0.35 Å^−1^ was also calculated for quantitative analysis of the edge-on PTB7 lamellar to face-on crystallite ratio^40^. The *I*_*out*_*/I*_*in*_ ratio of the SS-PTB7:PC_71_BM film was 3.96, whereas that of the SC-PTB7:PC_71_BM film was 29.1, indicating a significantly lower degree of edge-on orientation.

Enhanced crystallization and orientation of the molecules in the SS-PTB7:PC_71_BM film can affect the air stability of the film^41^,^42^,^43^. The mass density of an organic thin film, which is determined by the degree of crystallinity, can alter the ability of chemical reactants to permeate to the reaction site of molecules^41^. Enhanced molecular packing can also strengthen chemical bonds in the film due to more rigid and planar backbones, extending the regions of delocalization, leading to a chemically more robust film^42^,^43^.(Supplementary Note 7; Supplementary Fig. 8)

### Air processability

The air stability of the films formed by various processes was evaluated based on the Raman spectra of the SC- and SS-PTB7:PC_71_BM films obtained under 514 nm excitation. Raman peaks corresponding to phenylene-alkoxy (–R–O–) stretching (∼1,250 and ∼1,350 cm^−1^) and the C=C stretching mode of the BDT group (∼1,500, ∼1,550 cm^−1^ and ∼1,580 cm^−1^) were observed for the air-processed SC-PTB7:PC_71_BM film (Supplementary Note 8; Supplementary Fig. 9). The intensity of these peaks was significantly lower than that of the peaks of the N_2_-processed SC-PTB7:PC_71_BM film. This suggests that oxygen that infiltrated into the film during the spin-coating and drying processes contributes to cleavage of the alkoxy groups and insertion of oxygen into the C=C bonds in the backbone, resulting in disruption of the *π*-conjugation. This is consistent with the decreased and slightly blue-shifted absorption and photoluminescence of the air-processed SC-PTB7:PC_71_BM film (Fig. 2h,e).

A survey scan using X-ray photoelectron spectroscopy (XPS) was also performed to confirm the air stability of the SS films^44^,^45^. Figure 2i shows a considerable shift and peak broadening of the S2p peak for the air-processed SC-PTB7:PC_71_BM film, which can be ascribed to chain scissions in the polymer backbone, or to loss of the side chains. The C1s spectrum of the air-processed SC-PTB7:PC_71_BM film shows the emergence of a peak at 287 eV, derived from oxygenated carbon species, including a carboxyl group^13^. In contrast, no detectable S2p peak broadening or C1s peak from oxygenated carbon species was observed for the SS-BHJ and N_2_-processed SC-BHJ films (Fig. 2i).

**Figure 3:**
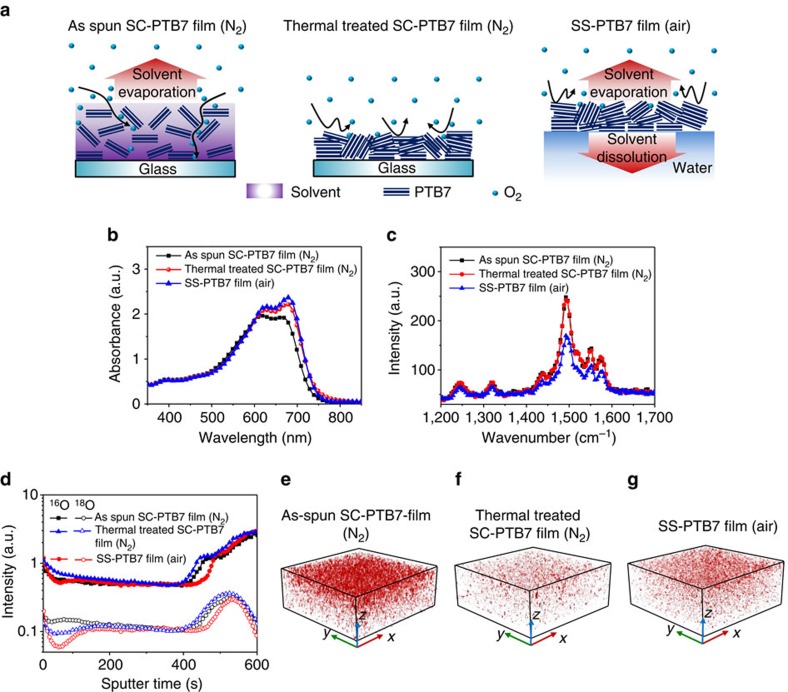
Oxygen diffusion into polymer films via solvent. (**a**) Oxygen diffusion mechanism in PTB7 films during drying process for spin-coated PTB7 films treated under different conditions and for SS-PTB7 film. (**b**) Ultraviolet–visible absorption spectra and (**c**) Raman spectra of spin-coated PTB7 films treated under different conditions, (**d**–**g**) TOF-SIMS depth profile and 3D iso-surface overlay of oxygen ions (^16^O and ^18^O) intensity as a function of sputter time for the spin-coated PTB7 films treated under different conditions.

Surprisingly, no indication of degradation was observed for the SS-PTB7:PC_71_BM films based on the XPS and Raman spectra (Fig. 2i; Supplementary Fig. 9) although the film was formed on water in air. For the air-processed SC-BHJ films, CB remaining between the polymer chains before complete drying may work as effective percolation pathways for diffusion of oxygen to the PTB7 molecules because of the significantly higher oxygen diffusivity of CB (1.16 × 10^−5^ cm^2^ s^−1^) than that of PTB7 (1.2 × 10^−8^ cm^2^ s^−1^)^46^,^47^, which can facilitate reactions between oxygen and the BDT group of PTB7 (Fig. 3a)^47^,^48^,^49^.

To examine the aftermath effects of remaining solvent on degradation of the BHJ films, the atomic ratio of oxygen to sulfur in the polymer films exposed to ambient conditions versus the drying time was probed by using XPS. Briefly, the BHJ films were spin-coated under N_2_ and subsequently exposed to air for different periods; finally, the films were completely dried in a N_2_-filled glove box (Supplementary Note 9; Supplementary Fig. 10a). The films exposed to air for a longer time exhibited greater oxygen permeation with a broad distribution of oxygen in the films, implying that the CB molecules could provide pathways for oxygen diffusion into the BHJ film, as presented in Fig. 3a. In contrast, the O/S ratio and the atomic distribution of oxygen in SS-PTB7:PC_71_BM film were comparable to that of the N_2_-processed SC-PTB7:PC_71_BM film, suggesting excellent air processability by the spontaneous spreading process (Supplementary Note 9; Supplementary Fig. 10b).

The effect of solvent (here, CB) on oxidation of polymer films was evaluated. DIO additive as a co-solvent was not considered because DIO molecules are steadily removed from films during the SS process due to greater specific gravity of DIO than water, as previously mentioned.(Supplementary Table 1, Supplementary Note 10 and Supplementary Fig. 11) For the experiments, PTB7 only (that is, without fullerenes) films were prepared using different post-treatments. PTB7 films spun in nitrogen were treated under different conditions (Fig. 3a) before exposure to air for 3 h, that is, (i) as-spun without removal of solvent, (ii) thermally treated at 70 °C for 20 min. After exposure to air for 3 h, the intensity of the absorption peaks of the as-spun PTB7 film with excess solvent were considerably reduced (Fig. 3b), indicating degradation of the PTB7 polymer chains due to air exposure, as compared with the thermally treated PTB7 films. Moreover, the Raman peaks of the as-spun PTB7 film obtained under excitation at 514 nm were also significantly diminished relative to those of the other films, suggesting cleavage of the alkoxy groups and insertion of oxygen into the C=C bonds in the backbone (Fig. 3c). Oxygen within the three PTB7 films was tracked using time-of-flight secondary ion mass spectrometry (TOF-SIMS), as shown in the depth profile in Fig. 3d. Figure 3e–g shows the 3D iso-surface overlay of the intensity of the oxygen ion peaks (^16^O and ^18^O). The intensity of oxygen near the surface of the as-spun PTB7 film was considerably higher than that of the thermally-treated PTB7 film (Fig. 3e–f), confirming that oxygen molecules penetrated the PTB7 film via the excess solvent between the PTB7 chains as explained above. In contrast, the oxygen intensity near the surface region of the SS-PTB7 film was comparable to that of the thermally treated film (Fig. 3f,g). In addition, performed studies on the effect of solvent on exposure to light and air on the oxidation of PTB7 films also suggest that oxygen adsorbed through the solvent path in the film during illumination in air contributes to accelerated disruption of the backbone conjugation of BDT group in PTB7 molecules. (Supplementary Note 11; Supplementary Fig. 12)

It can be inferred that the spontaneous spreading process can induce rapid formation of a condensed and well-packed thin film due to the ultrafast solvent removal, which can prevent the intrusion of oxygen via the CB solvent, impeding oxidation and redistribution of the polymer and PC_71_BM molecules in the BHJ film.

### Photovoltaic performances

**Figure 4:**
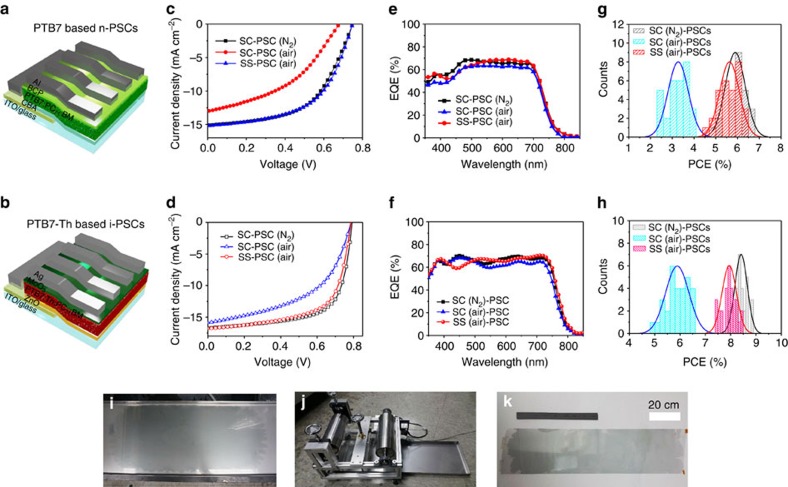
Fabrication of organic solar cells using SS films and potential for scalable process. (**a**,**b**) Device structure of n-PSC (PTB7:PC_71_BM) and i-PSC (PTB7-Th:PC_71_BM); (**c**–**f**) *J–V* characteristics and EQE spectra of PTB7-based (**c**,**e**) and PTB7-based PSCs (**d**,**f**) under AM 1.5G 100 mW cm^−2^ illumination. (**g,h**) statistics for PCEs of 30 samples. (**i**) Formation of a single SS film on a large water bath (30 × 120 cm^2^). (**j**) Homemade R2R coating system for large-scale transfer on flexible substrates. (**k**) 25 × 100 cm^2^ PET film coated with a SS-PTB7:PC_71_BM film.

Polymer solar cells (hereafter, SS-PSCs) were fabricated to evaluate the applicability of the SS films to high-efficiency functional devices fabricated in air. For comparison with the SS-PSCs, SC-PSCs were fabricated under two different sets of environmental conditions, that is, in a N_2_ filled glove box (hereafter, SC-PSCs (N_2_)) and under ambient atmosphere (hereafter, SC-PSCs (air)). The air exposure time of the SC-PSCs (air) and SS-PSCs during the coating process in air was set to under 10 min. The post-drying process for the SC-PSCs (air and N_2_) was performed in a N_2_ filled glove box (see Methods section). The device structure (Fig. 4a,e) was ITO/CBA (or ZnO)/ PTB7 (or PTB7-Th):PC_71_BM/BCP/Al (or MoO_3_/Ag).

Figure 4b,f show the representative current density–voltage (*J*–*V*) characteristics of the SS-PSCs, SC-PSCs (N_2_) and SC-PSCs (air) based on a mixture of PTB7:PC_71_BM and PTB7-Th:PC_71_BM. The SC-PSCs (air) exhibited much lower performance than the SS-PSCs and SC-PSCs (N_2_), attributed to degradation of the air-processed BHJ films by oxidization as explained in relation to Fig. 2f.

The PCE of the PTB7-based SS-PSC was 6.27% (Table 1, Supplementary Note 12 and Supplementary Table 2), which is comparable to that of the SC-PSC (N_2_; 6.21%). However, the PCE of the SC-PSCs (air) was significantly reduced (3.74%). The PCE of the PTB7-Th-based SS-PSC reached 8.44%, and this is the highest PCE reported for air-processed organic solar cells to date to the best of our knowledge. Details of the device performance are summarized in Table 1.

Both polymer-based SS-PSCs exhibited performance comparable to that of the SC-PSCs (N_2_) with an almost identical spectral response in the external quantum efficiency (EQE) data (Fig. 4c,g). To evaluate the reliability of the SS-PSCs processed in air, statistical data for the PCEs of 30 PTB7 and PTB7-Th devices are presented in Fig. 4d,h and Supplementary Figs 13 and 14 with Supplementary Note 13, respectively.

We demonstrated the potential of our method as a scalable process by transferring the SS film to a large PET substrate using an R2R process. Figure 4i shows a SS -PTB7:PC_71_BM film on the water bath (30 × 120 cm^2^ size), made by dropping only 200 μl of the polymer solution as a point source for spontaneous spreading (Supplementary Movie 2). After the solution spread, a 1 m long SS film was obtained in a few seconds. A custom-made R2R system was used to transfer the large SS film to the PET substrate (Fig. 4j). The SS-film was transferred successfully from the water surface to the PET substrate (Supplementary Movie 3). Figure 4k shows a PET film coated with a PTB7:PC_71_BM layer ∼60 nm thick. We anticipate that this new polymer film formation method will have great potential as a new process for R2R-processed modules with low material consumption and drying time, and consequently lower fabrication cost.

In summary, we developed an air-processable technique to form polymer films on an aqueous surface within a few seconds, using the spontaneous spreading phenomenon. Ultrafast removal of solvent during the process causes the films to have uniform and high-quality nanomorphology because oxygen diffuses into the polymer films rapidly through the remaining solvent between the polymer chains. Furthermore, the polymer films were successfully transferred onto various substrates. Finally, we demonstrated high performance of PSCs prepared using the proposed process, comparable to that of PSCs prepared by spin coating. We expect that this approach can be extended by roll-to-roll production to achieve high-quality films.

## Methods

### Preparation of the polymer solution

For the SC films, PTB7:PC_71_BM (1-Materials: Nano-C) and PTB7-Th:PC_71_BM (1-Materials: Nano-C) at a weight ratio of 1:1.5 were dissolved in chlorobenzene (CB):1,8-diiodoctane (DIO) (97:3 v/v) at 50 °C. This mixture was stirred for 12 h under N_2_. For the SS-films, PTB7:PC_71_BM and PTB7-Th:PC_71_BM at a weight ratio of 1:1.5 were dissolved in 10 vol% of DIO in CB (CB:DIO=90:10 v/v).

### Solar cell fabrication for PTB7:PC_71_BM

PTB7:PC_71_BM PSCs were fabricated on ITO-deposited glass substrates (*R*_sh_: 22 ohm sq^−1^). A layer of 4-chlorobenzoic acid (CBA, Sigma-Aldrich) as a hole transfer layer (HTL) was spun onto the substrates at 3,000 r.p.m. for 30 s; the substrates were then annealed at 70 °C for 5 min (ref. 50). Surplus molecules of CBA on the substrates were removed by spin coating with methanol at 6,000 r.p.m. for 30 s. Two layers of SS-PTB7: PC_71_BM films were transferred sequentially onto the substrates and dried at 70 °C for 10 min under N_2_. SC-PTB7: PC_71_BM films were spun at 2,500 r.p.m. for 30 s and then dried at 70 °C for 10 min under N_2_. Subsequently, bathrocuproine (BCP, 8 nm) and Al (150 nm) were deposited through a shadow mask by thermal evaporation onto the devices^51^.

### Solar cell fabrication for PTB7-Th:PC_71_BM

PTB7-Th:PC_71_BM PSCs were fabricated on the same ITO glass substrates with PTB7:PC_71_BM PSCs. A layer of ZnO for electron transfer layer (ETL) was spun onto the substrates at 6,000 r.p.m. for 30 s, and the substrates were then annealed at 200 °C for 20 min (ref. 25). Two layers of SS-PTB7:PC_71_BM films were transferred sequentially onto the substrates. The SC-PTB7: PC_71_BM films were spun at 2,000 r.p.m. for 30 s. There was no additional drying process or annealing. Subsequently, MoO_3_ (9 nm) and Ag (150 nm) were deposited through a shadow mask by thermal evaporation onto the devices^52^.

### Measurement of polymer solar cells

The current density–voltage (*J*–*V*) characteristics of the cells were measured from 1.0 to −0.2  V using a solar simulator (K201 LAB55, McScience) under irradiance of 100 mW cm^−2^ from a 150 W Xe short-arc lamp filtered by an air mass 1.5 G filtre. Light intensity was calibrated with a Si reference cell (McScience, K801S-K302). The voltage scan rate was 100 mV s^−1^ and no device preconditioning was applied before starting the measurement, such as light soaking. A 6.25 mm^2^ aperture mask was attached to the device to define the illuminated area clearly. The quantum efficiency (QE) spectra of the solar cells were obtained using a spectral (K3100 IQX, McScience Inc.) measurement system. The integrated response (*J*_*SC*_) under the standard reference spectrum was compared with the *J*_*SC*_ value measured under the simulator, and they showed an ∼2% *J*_*SC*_ difference.

### Film analysis

The surface morphology of the BHJ films was observed using atomic force microscopy in tapping mode under ambient conditions (AFM, Nanoman, Veeco) using a field emission scanning electron microscope (FE-SEM, FEI Sirion) and a field emission transmission electron microscope (FE-TEM, FEI Tecnai G2 f30 S-Twin, 300 KeV). Photoluminescence quenching was evaluated for the SC- and SS-PTB7:PC_71_BM films without metal electrodes using a Horiba Jobin Yvon NanoLog spectrophotometer. We performed the measurement with at least three samples to reduce the sample-to-sample variation and measurement error. The XPS and ultraviolet photoelectron spectroscopy spectra were recorded with a Sigma Probe (Thermo VG Scientific) instrument, using a micro-focused monochromatic Al X-ray source with a beam energy of 1468.74 eV and a takeoff angle of 45°. Raman spectra were obtained using a micro-Raman system (LabRam HR spectrometer, JY Horiba). A 514 nm solid-state laser and a 633 nm He–Ne laser were used as the excitation light sources and were kept to less than 0.5 mW to prevent photo-degradation of the BHJ films. The laser was focused to a beam diameter of ∼5 μm on the sample using a × 50 objective microscope lens. The Raman spectra were background corrected and obtained by averaging the spectra obtained from three different regions of each sample. The spectra from the different regions were almost the same, demonstrating the reliability of the results. We also confirmed the outcomes by measuring three samples prepared at different times. The spectral resolutions are within 0.4 and 0.2 cm^−1^ for the 514 and 633 nm excitations, respectively. GIWAXS measurement was performed using X-rays with a wavelength of *λ*=1.54 Å at the 8C1 beam line of the Pohang Accelerator Laboratory. The 3D profiles for oxygen distribution in the PTB7 films were obtained using a TOF-SIMS 5 system (ION-TOF GmbH, Münster, Germany) equipped with a 30 KeV liquid metal ion gun and a time-of-flight mass spectrometer. To sputter the PTB7 film, a 1 KeV beam of Cs^+^ ions was used to remove material by sputtering a 300 μm × 300 μm region, whereas a 30 KeV beam of Bi^3+^ ions was employed to analyse the composition distribution by acquiring the mass-to-charge ratios (*m/q*) of the negatively charged secondary ions, for example, C^−^ (*m*/*q*=12), S^−^ (*m*/*q*=32), ^16^O^−^ (*m*/*q*=16), ^18^O^−^ (*m*/*q*=16) from the analysis area (100 μm × 100 μm) centered at the sputtered region. Data acquisition and subsequent data processing and analysis were performed using SurfaceLab 6 software (ION-TOF GmbH).

### Data availability

The authors declare that the data supporting the findings of this study are available within the article and its Supplementary Information.

## Additional Information

**How to cite this article:** Noh, J. *et al*. Ultrafast formation of air-processable and high-quality polymer films on an aqueous substrate. *Nat. Commun.* 7:12374 doi: 10.1038/ncomms12374 (2016).

## Supplementary Material

Supplementary Information Supplementary Figures 1-14, Supplementary Tables 1-2, Supplementary Notes 1-13 and Supplementary References.

Supplementary Movie 1 Formation of SS-PTB7:PC71BM film on water surface. A small portion of the film was transferred to a glass substrate.

Supplementary Movie 2 Formation of a 1 m long film for the roll-to-roll process.

Supplementary Movie 3 Demonstration of large-area, continuous transfer using roll-to-roll production system.

## Figures and Tables

**Table 1:** Representative photovoltaic performance parameters for SC- and SS-PSCs.

**PSCs**	**Performance**	**SC-PSC (N**_**2**_)	**SC-PSC (air)**	**SS-PSC (air)**
PTB7:PC_71_BM	PCE (%)	5.90±0.27 (6.34)	3.32±0.25 (3.74)	5.84±0.25 (6.24)
	*J*_sc_ (mA cm^−2^)	14.22±0.83 (15.71)	12.27±0.67 (13.28)	14.71±0.57 (15.45)
	*V*_oc_ (V)	0.74±0.03 (0.77)	0.65±0.03 (0.69)	0.74±0.01 (0.76)
	FF	0.56±0.03 (0.60)	0.42±0.02 (0.46)	0.54±0.02 (0.57)
				
PTB7-Th:PC_71_BM	PCE (%)	8.41±0.24 (8.86)	5.89±0.46 (6.29)	7.95±0.28 (8.44)
	*J*_sc_ (mA cm^−2^)	16.80±0.27 (16.72)	14.71±0.84 (15.85)	16.66±0.34 (16.80)
	*V*_oc_ (V)	0.80±0.01 (0.79)	0.78±0.01 (0.79)	0.79±0.01 (0.79)
	FF	0.63±0.02 (0.67)	0.51±0.03 (0.50)	0.60±0.02 (0.64)

PSCs, polymer solar cells; PTB7, poly[[4,8-bis[(2-ethylhexyl)oxy]benzo[1,2-b:4,5-b']dithiophene-2,6-diyl][3-fluoro-2-[(2-ethylhexyl) carbonyl]thieno[3,4-b]thiophenediyl]]; SS, spontaneously spreading.

Means and s.d. are obtained from 30 devices. The values in brackets illustrates the best cell performance for each device.
