# Supplementary material for: Ultrafast formation of air-processable and high-quality polymer films on an aqueous substrate
Source: Nat Commun. 2016 Aug 10;7:12374. doi: 10.1038/ncomms12374 (PMC4987522; doi:10.1038/ncomms12374)
Supplement: Supplementary Information — Supplementary Figures 1-14, Supplementary Tables 1-2, Supplementary Notes 1-13 and Supplementary References. [file ncomms12374-s1.pdf]

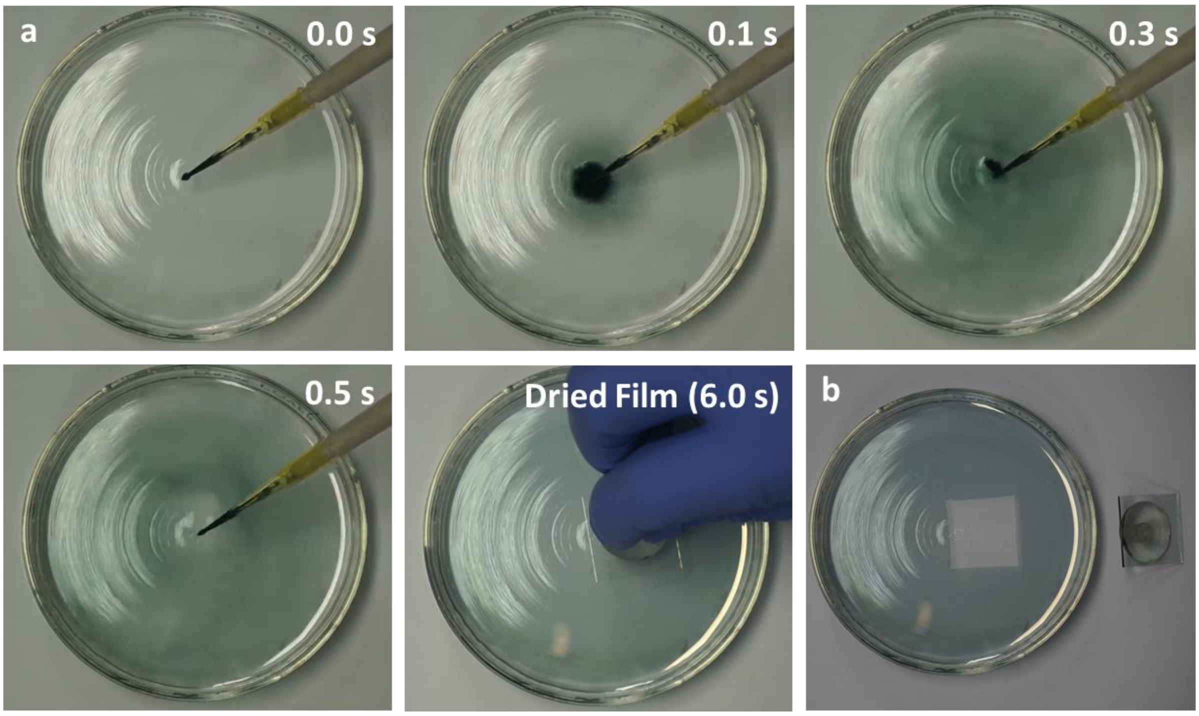

Supplementary Figure 1. a, Snapshots of spontaneous spreading process for polymer solution on water surface, **b,** The polymer films can be transferred to target substrates easily.

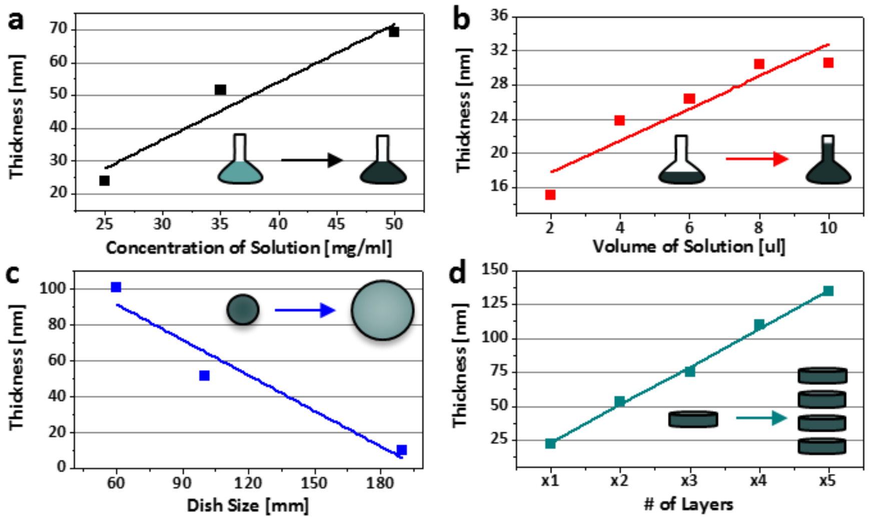

Supplementary Figure 2. Variation of the thickness of the SS films with variation of the concentration of the solution **(a)**, volume **(b)**, bath size **(c)**, and the stacking number of the films **(d)**.

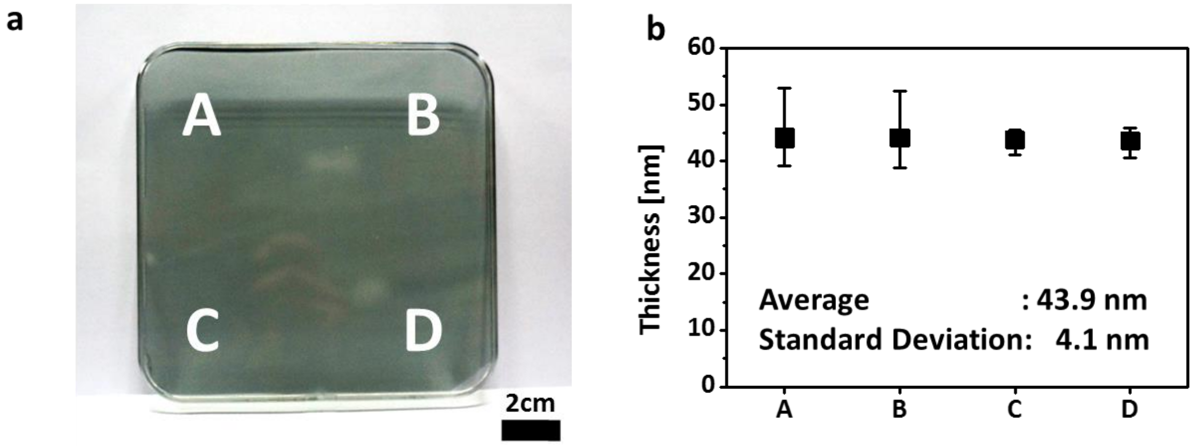

Supplementary Figure 3. a, Large area deposition of SS film on 12 cm × 12 cm Petri dish, **b,** thickness of the film measured at four corners: A, B, C and D.

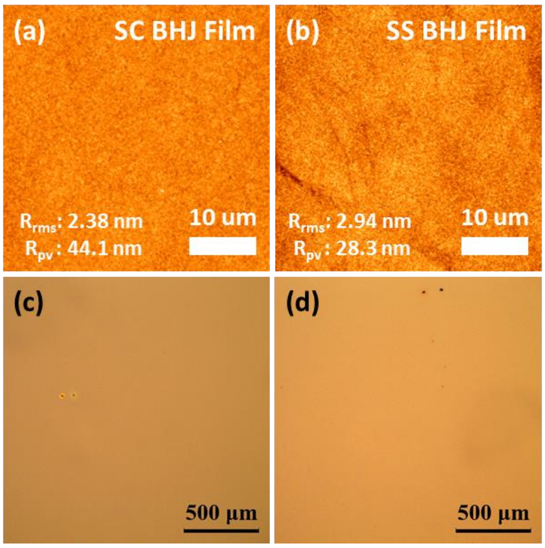

Supplementary Figure 4. a-b, AFM images (40 µm x 40 µm) of (a) SC- and (b) SS- PTB7:$PC_{71}BM$ BHJ films. c-d, Optical microscopic images of (c) SC- and (d) SS- BHJ films.

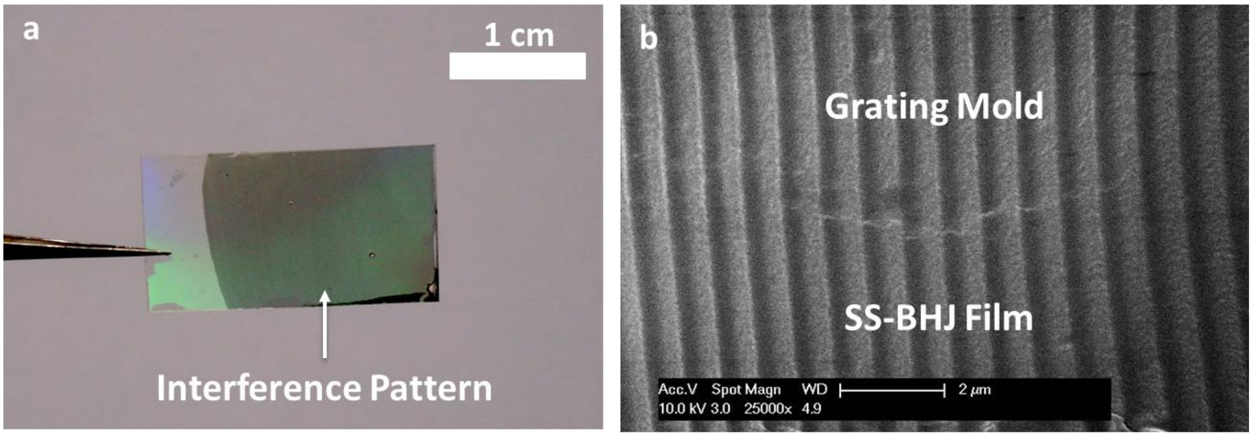

Supplementary Figure 5. a, Transferred SS films on polyurethane substrate patterned with 556 nm sinusoidal grating; interference pattern shows that the film followed the nano-pattern. **b,** SEM image shows conformally deposited SS-BHJ film on the grating mold.

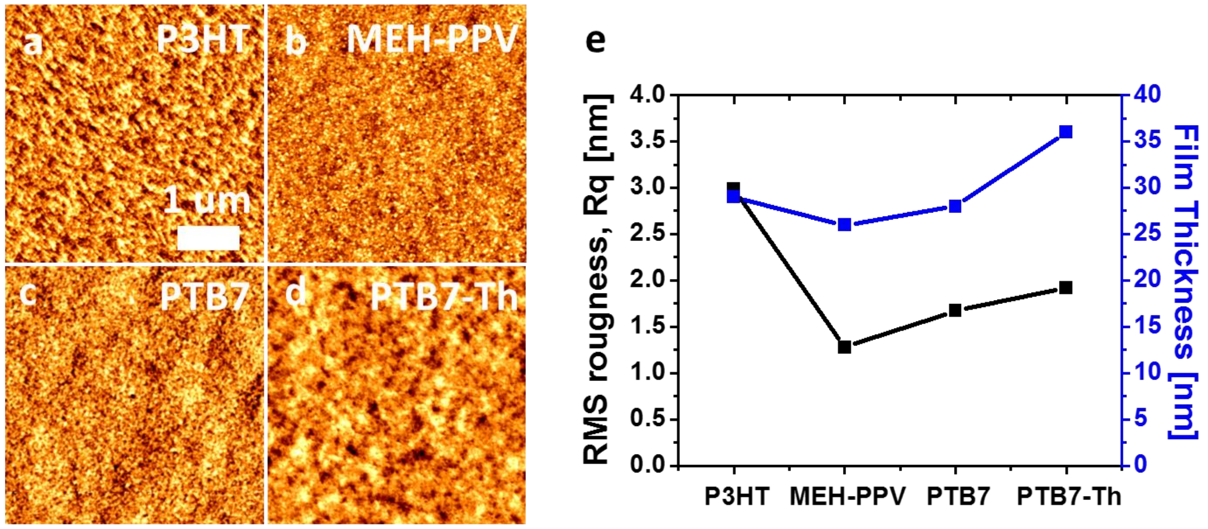

Supplementary Figure 6. a-d, AFM images of SS films from several types of polymers: (**a**) P3HT, (**b**) MEH-PPV, (**c**) PTB7, and (**d**) PTB7-Th. **e,** RMS roughness and thickness data for the SS films.

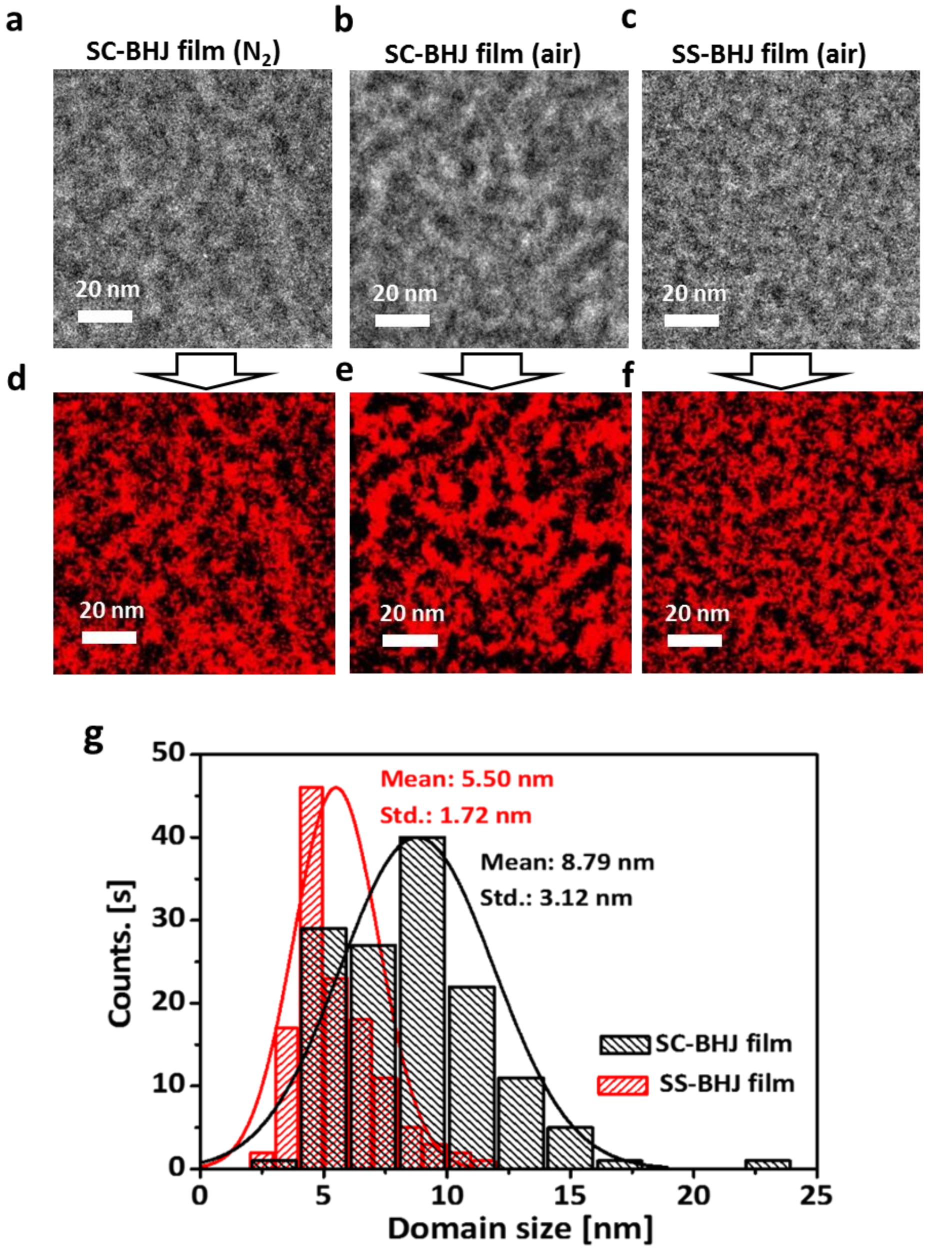

Supplementary Figure 7. **a-c,** TEM images and **d-f,** colored images (red: PTB7 domains) measured at the same defocusing distance for (**a** and **d**) N_2 and (**b** and **e**) air-processed SC-BHJ, and (**c** and **f**) SS- BHJ films. **g**, size distribution histograms of PTB7 domains within N_2-processed SC-BHJ and SS-BHJ films.

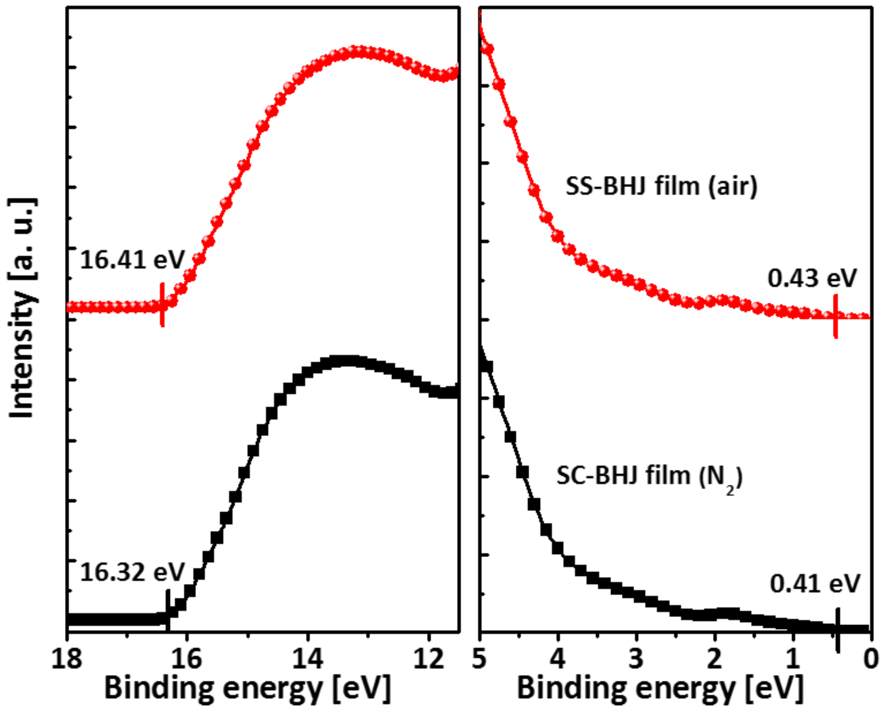

Supplementary Figure 8. a, b, UPS spectra of **(a)** the secondary edge region and **(b)** the HOMO region.

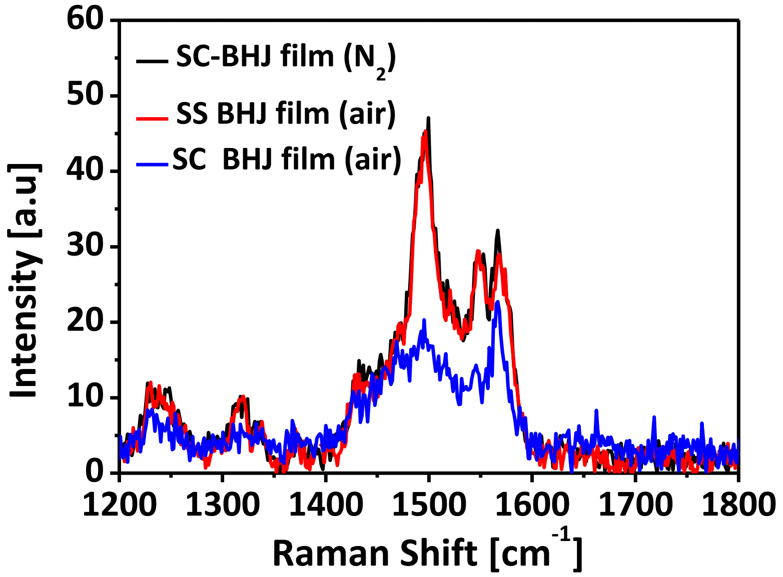

Supplementary Figure 9. Raman spectra of SC-PTB7:PC$_{71}$BM films processed in air and N_2 and of the SS-PTB7:PC$_{71}$BM film under 514 nm excitation.

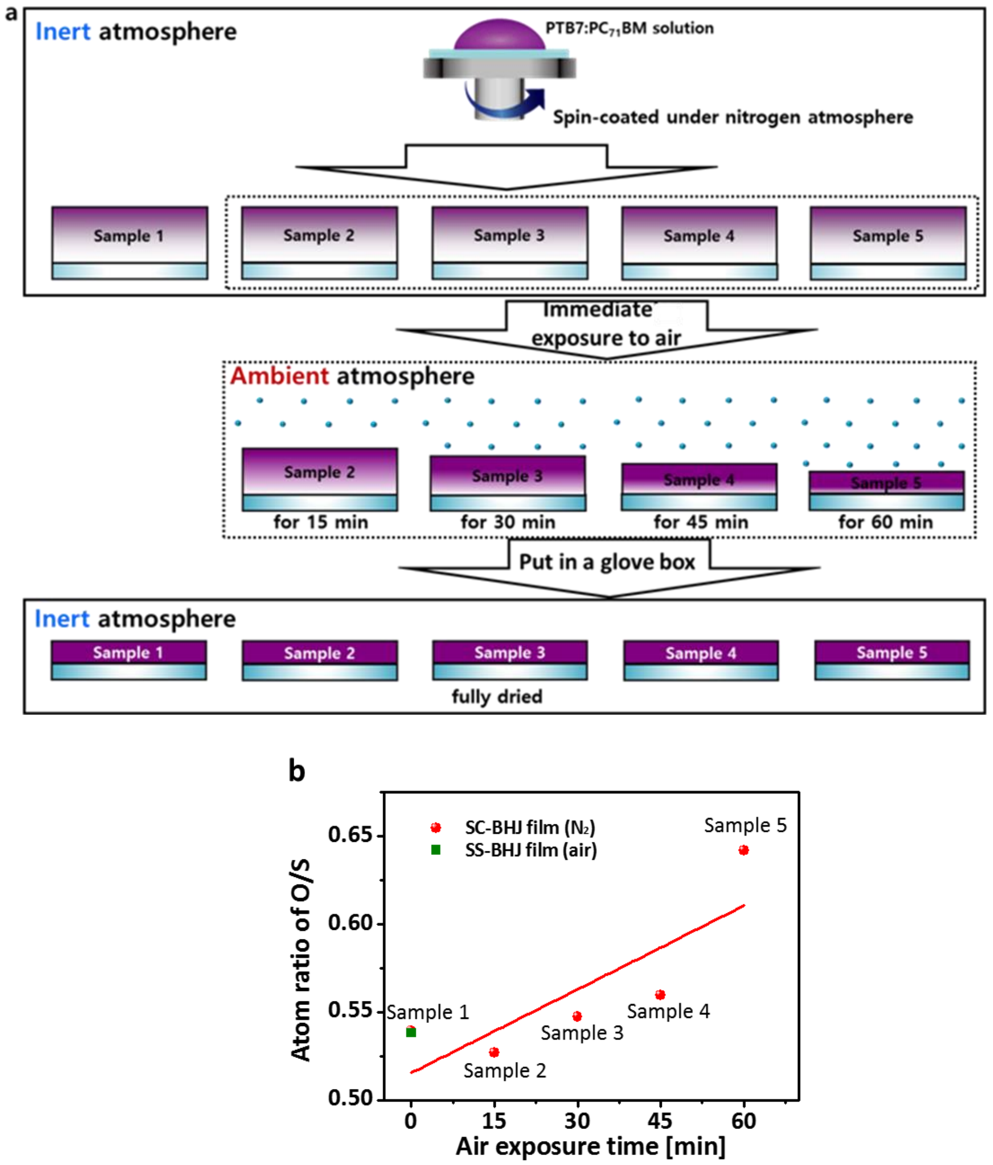

Supplementary Figure 10. a, Schematic illustration of the oxidation of SC-BHJ films processed under N_2 and exposed to air for different times, and **b,** the atomic ratio of oxygen to sulfur (O/S) in the SC-BHJ films after air exposure as a function of drying time and in as-deposited SS-BHJ film. The data were obtained by XPS.

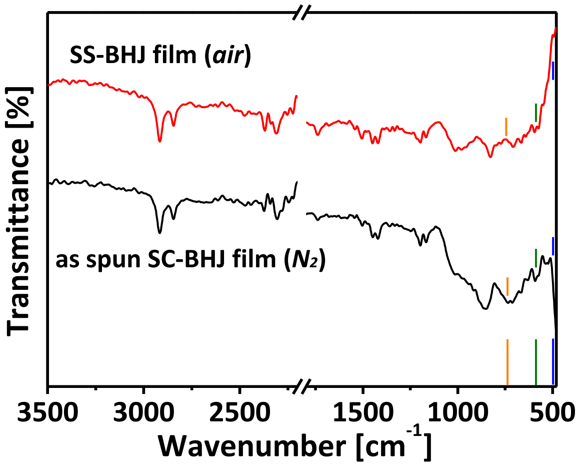

Supplementary Figure 11. FT-IR spectra of the as-spun PTB7 film in N_2 and the SS-BHJ film.

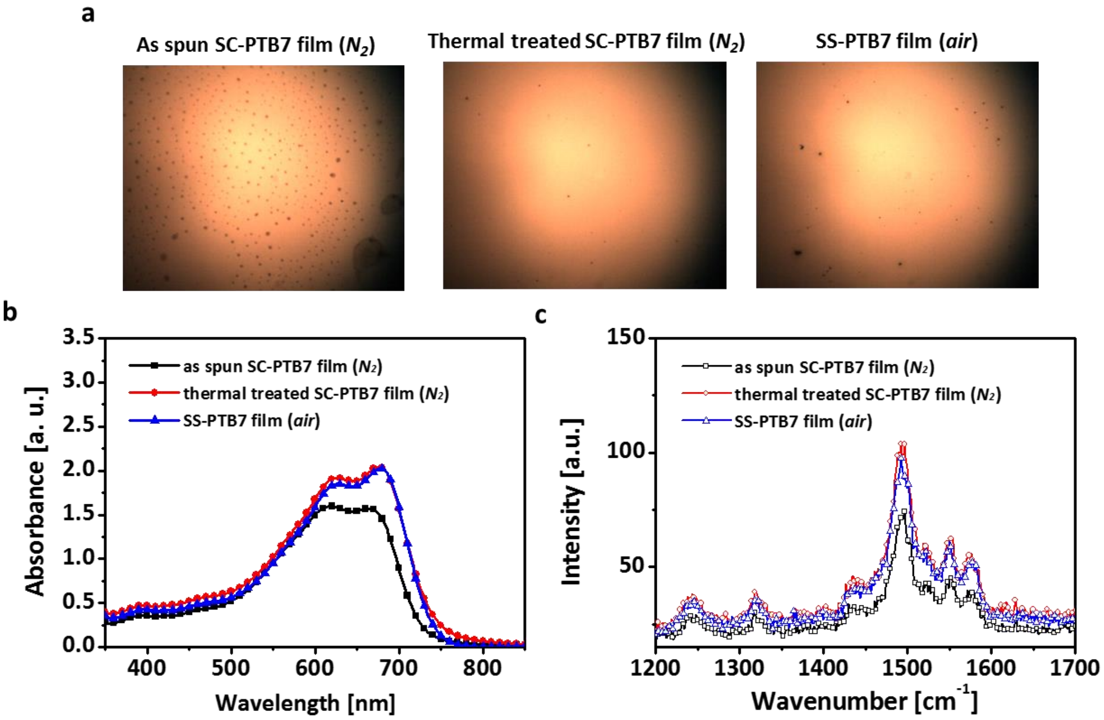

Supplementary Figure 12. a, optical images of the spin-coated PTB7 films treated under different conditions and SS-PTB7 film after 1h of illumination in air **b**, UV-vis. absorption spectra, and **c**, Raman spectra for the spin-coated PTB7 films treated under different conditions.

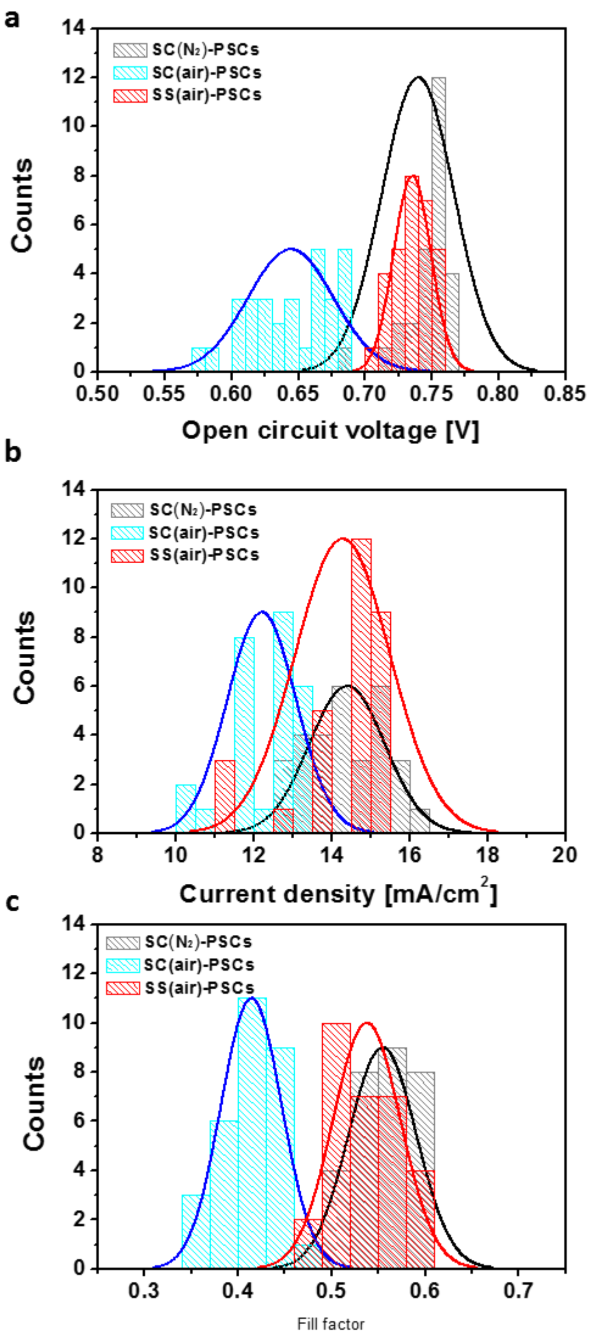

Supplementary Figure 13. a-c, Statistical data for air and N_2 processed SC- and SS-PSCs based on PTB7: V_{oc} **(a)**, J_{sc} **(b),** and *FF* **(c)**.

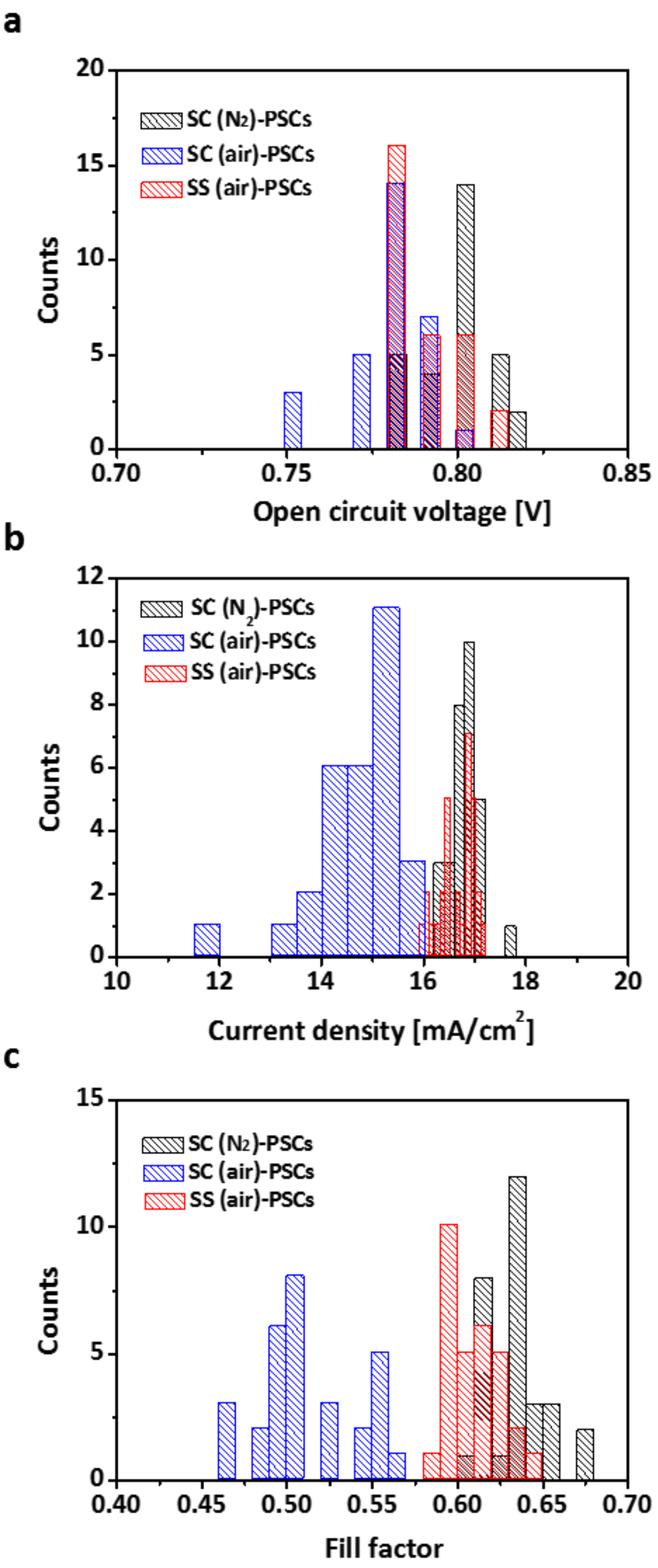

Supplementary Figure 14. Statistical data for air and N_2 processed SC- and SS-PSCs based on PTB7-th: V_{oc} **(a)**, J_{sc} **(b),** and *FF* **(c)**.

Supplementary Table

Supplementary Table 1. Physical and chemical properties of solvents

**Solvent**	**Water**	**Chlorobenzene**	**1,8-Diidooctane**
**surface tension at 25 ˚C (dyne cm^{-1})**	72.86 ± 0.05	33.1 ± 3.0	40.0 ± 3.0
**Specific gravity/Density (g cm^{-3})**	1	1.1	1.84
**Water Solubility at 25 ˚C (mg L^{-1})**	-	392.21	0.2301
**Boiling point (˚C)**	100	131	167-169

Supplementary Table 2. Characteristics of devices for SC (DIO 3%) and SS with different DIO concentration

**PSCs**	**Performance**	**SC (DIO: 3%)**	**SS (DIO: 2%)**	**SS (DIO: 3%)**	**SS (DIO: 4%)**	**SS (DIO: 6%)**	**SS (DIO: 10%)**
**PTB7 :$PC_{71}BM$**	**PCE [%]**	6.20±0.02 (6.22)	2.94±0.23 (3.21)	4.22±0.35 (4.63)	5.22±0.05 (5.29)	5.34±0.07 (5.41)	6.08±0.05 (6.15)
	**J_{sc} [mA cm^{-2}]**	13.79±0.04 (13.79)	11.00±0.45 (11.55)	13.11±00.25 (13.27)	14.13±0.09 (14.23)	15.12±0.09 (15.23)	15.27±0.08 (15.31)
	**V_{oc} [V]**	0.757±0.003 (0.762)	0.671±0.005 (0.679)	0.663±0.015 (0.679)	0.679±0.004 (0.684)	0.734±0.014 (0.743)	0.737±0.011 (0.756)
	FF	0.59±0.00 (0.59)	0.40±0.01 (0.41)	0.48±0.03 (0.51)	0.54±0.00 (0.55)	0.48±0.00 (0.49)	0.54±0.00 (0.54)

Means and standard deviations are obtained from 30 devices. The value in brackets illustrates the best cell performance for each device.

Supplementary Note 1| In-situ observation of spontaneous spreading and transfer

Supplementary Figure 1 shows that a polymer film was formed and dried within a few seconds on water after spontaneous spreading, and that the film could easily be transferred to a target substrate.

Supplementary Note 2| Variation of the thickness of the SS films

The area covered by the dripped solution before it dries is mainly determined by the *S* value when the bath is large enough. Hence, the concentration of the polymer solution is proportionally related to the thickness of the polymer film for a given volume of the solution. The thickness of the SS films formed on water with 25 and 35 mg mL^{-1} of polymer concentration are 24.1 and 51.6 nm, respectively. The film thickness increased almost linearly up to 69.3 nm with 50 mg mL^{-1} of polymer when the volume of the drop was 10 μL (bath diameter: 100 mm), as shown in Supplementary Figure 2a. A higher density of solution contains a larger amount of polymer and therefore results in thicker polymer films. It should be emphasized that no organic materials are wasted because they stay on the water after the solvent dries or permeate into the water.

Figure 2b shows the relation between the film thickness and volume of the solution (25 mg mL^{-1}). Shortly after a drop of the polymer solution makes contact with the water surface, the solution spreads to cover the entire surface of the water in a Petri dish (diameter: 100 mm) by Marangoni flow. The thickness of the film is proportional to the volume of the blend, as shown in Supplementary Figure 2b. The SS-film thickness was approximately 30 nm when the volume of the solution was 8 μL. If the solvent is not dried fully within a few seconds, the thin liquid layer remaining attempts to assume an equilibrium contact angle by aggregating at multiple points to form discrete droplets on the water surface. Consequently, even if too large a volume of the polymer solution is used, there is a limit to how thick the SS film can become.[1]

The area of the spontaneous spreading films can also be limited by the size of the water bath. Assuming the same volume and density of solution, the film thickness would decrease as the bath size increases. Supplementary Figure 2c shows the thickness of the SS film with respect to the size of the water bath. A 10 μL drop of the solution (35 mg ml^{-1}) formed SS films of 101.3, 51.6, and 10.0 nm in baths with diameters of 60, 100, and 190 mm, respectively.

Although the thickness of a single SS film is limited to approximately 70 nm, SS films could be transferred multiple times to another substrate to increase the overall thickness. For instance, a drop of PTB7:$PC_{71}BM$ (25 mg mL^{-1}, 10 μL) produced a 24 nm thick single SS film, and the total thickness of the multi-film increased linearly upon addition of a number of stacked films, as demonstrated in Supplementary Figure 2d.

Supplementary Note 3| Film uniformity over large area

We deposited a SS-PTB7:$PC_{71}BM$ film in a square PET petri dish (12 × 12 cm^2) and measured the thickness of 4 points in each corner area (total 16 positions). The average thickness of the SS film was 43.9 nm and the standard deviation was 4.1 nm. We chose the PET substrate as a representative example of large area transfer of SS films; however, the substrate materials are not limited to PET (Fig. 1c-f)

AFM and microscopic images of SC- and SS- PTB7:$PC_{71}BM$ BHJ films reveal the film quality in the large scale from micrometer to millimeter. (Supplementary Fig. 4) As described in the original manuscript, dewetting phenomenon, which causes pin holes in the liquid films, becomes widespread especially when the spreading coefficient is negative (S <0).[1,2] Because our SS film forms when S>0, the thin polymer film does not create pin holes during short drying time of a few seconds.

Supplementary Note 4| Transfer of spontaneous spreading (SS) film to various substrates

The SS films can be formed conformally on nano-patterned or rough surfaces. Supplementary Figure 5a shows that an interference pattern was still apparent on the polyurethane (PU) substrate with sinusoidal gratings (period: 556 nm) after the SS films were transferred to the substrate. Scanning electron microscope (SEM) images also confirmed successful formation of the SS films (Supplementary Fig. 5b).

Supplementary Note 5| Wide materials selection for SS film formation

Because the SS phenomenon occurs based on the interplay between solvents and substrates, the SS film formation process can be applied to various polymers. We demonstrated the SS film formation using poly(3-hexylthiophene-2,5-diyl) (P3HT), poly[2-methoxy-5-(2-ethylhexyloxy)-1,4-phenylenevinylene] (MEH-PPV), PTB7, and PTB7-Th (Supplementary Fig. 6). Upon dropping 20 µl solutions (10 mg ml^{-1}), SS films were similarly formed with a thickness of 26 ~ 36 nm and RMS roughness of 1.2 ~ 3 nm.

Supplementary Note 6| High-resolution transmission electron microscopy (HRTEM) images

HRTEM images of the SS-PTB7:$PC_{71}BM$ and SC-PTB7:$PC_{71}BM$ films (air and N_2) were obtained at the same defocusing distance to compare the domain sizes of PTB7, as shown in Supplementary Figure 7a-c. To visualize the $PC_{71}BM$ cluster domains (dark regions) and PTB7 crystallite domains

(bright regions) more clearly, we colored the bright regions in red, as shown in Supplementary Figure 7d-f.

For the SS-PTB7:$PC_{71}BM$ film, the average size of the PTB7 domains is distinctively smaller than that of the SC-PTB7:$PC_{71}BM$ films (air and N_2). For the SS-PTB7:$PC_{71}BM$ film, the average size of the PTB7 domains is 5.50 nm, while the SC-PTB7:$PC_{71}BM$ films show relatively larger PTB7 domains with an average size of 8.79 nm, as shown in Supplementary Figure 7g. We used at least 5 samples each, and the results were obtained from at least 25 PTB7 domains in each TEM image using ImageJ software.

Supplementary Note 7| Ultraviolet photoelectron spectroscopy (UPS) analysis

Supplementary Figure 8 shows the UPS spectra of the N_2-processed SC- and SS-PTB7:$PC_{71}BM$ films on a Si wafer. The HOMO energy levels were determined by using the following equation:

$$E_{HOMO} = h\nu - (E_{cutoff} - E_{onset}), \quad (1)$$

where $h\nu$ is the incident photon energy of He I (21.2 eV), E_{cutoff} is the high binding energy of the N_2-processed SC- and SS-PTB7:$PC_{71}BM$ films, which was determined by linear extrapolation to zero of the yield of secondary electrons, and E_{onset} is the onset energy of the N_2-processed SC- and SS-PTB7:$PC_{71}BM$ films relative to the Fermi level of Au.

The HOMO energy levels of the N_2-processed SC- and the SS-PTB7:$PC_{71}BM$ films were calculated to be 5.29 eV and 5.22 eV, respectively. The upward shift of the HOMO level can be attributed to morphological changes due to improved crystallization of the polymer phase and the subsequent change in the band-gap of the polymer.[2]

Supplementary Note 8| Raman spectra

Peaks at ~1250 cm^{-1} and ~1350 cm^{-1} correspond to the phenylene-alkoxy (-R-O) stretching mode, and those at ~1500, 1550, and ~1580 cm^{-1} to the C=C stretching mode of the benzodithiophene (BDT) group. The air-processed SC-PTB7:$PC_{71}BM$ film showed significantly decreased Raman intensities for these peaks, as compared with those of the N_2-processed SC-PTB7:$PC_{71}BM$ and the air-processed SS-PTB7:$PC_{71}BM$ films.

Supplementary Note 9| Correlation between the remaining solvent and the air stability of the polymer films

The correlation between the remaining solvent and the air stability of the polymer films was investigated by estimating the amount of oxygen in the films depending on the air-exposure time during film formation. For the experiment, the SC-BHJ films were prepared under N_2; subsequently, the films were exposed to air for different times; finally, the films were completely dried in a N_2-filled glove box, as depicted in Supplementary Figure 10a. The atomic ratio of oxygen to sulfur (O/S) in the SC-BHJ films as a function of the drying time in air is displayed in Supplementary Figure 10b. The volume created by excess solvent molecules could serve as pathways for oxygen diffusion into the BHJ films.

Supplementary Note 10| Fourier-transform infrared spectroscopy (FT-IR)

Although DIO evaporates slower than CB molecules, and water solubility of DIO is very low (water solubility at 25°C: 0.2301 mg L^{-1}), DIO molecules are steadily removed from films during the SS process, because of greater specific gravity (1.84 gcm^{-3}) of DIO than water (1 g cm^{-3}).(Supplementary Table 1)

We verified little DIO molecules are left within the BHJ film after the SS process from FT-IR measurements, as shown in Supplementary Figure 11. For FT-IR measurement, as-spun PTB7 film in N_2 without removal of solvents (CB and DIO) and the SS-BHJ film were prepared. Characteristics peaks at 720, 595, and 505 cm^{-1} are attributed to C-I stretching vibration of DIO molecules.[3] As shown in Supplementary Figure 10, the DIO peaks are clearly observed in as-spun PTB7 film, but no clear peak can be found in the SS-BHJ film, suggesting DIO solvent was effectively removed during the SS process on the water surface.

Supplementary Note 11| The effect of solvent upon exposure to light and air on the oxidation of PTB7 films

We additionally studied the effect of solvent upon exposure to light and air on the oxidation of PTB7 films. Prior to light and air exposure for 1 h, the spin-coated PTB7 films in N_2 –filled glove box were treated under different conditions to remove solvent; i) as-spun without removal of solvent, ii) thermally treated at 70 °C for 20 min. Refer to Supplementary Figure 12a.

The optical images show that as-spun PTB7 film with excess solvent is inhomogeneous with many aggregates after 1h of illumination in air, differently from two films without solvents, as shown in Supplementary Figure 12a. Furthermore, similar to the films exposed to air only (see Figure 3b), absorption of the as-spun PTB7 film with excess solvent were significantly decreased and hypochromic shift was observed in the absorption peaks at 625 nm and 680 nm, indicating the

disruption of the backbone conjugation of BDT group in PTB7 molecules with air and light exposure, as compared with the rest, as shown in Figure Supplementary Figure 12b.

In Raman spectra of PTB7 films obtained under 514 nm excitation (Supplementary Figure 12c), peaks from BDT group (C=C) for the as-spun PTB7 film (1499 cm^{-1}) were significantly lower and shifted than those of SC-PTB7 (1489 cm^{-1}) and SS-PTB7 (1489 cm^{-1}) films after 1h of illumination in air. Furthermore, the relative intensity of peaks at ∼1535 and ∼1575 cm^{-1} due to fused thiophene and benzene group increased. These suggest that oxygen adsorbed through the path providing solvent molecules in the film during illumination in air contributes to accelerated cleavage of alkoxy groups and insertion of oxygen into C=C bonds in the backbone.

Supplementary Note 12| The performance of SS-PSCs with various DIO concentrations

Performances of SS-PSC devices fabricated with various DIO concentrations (2, 3, 4, 6 and 10 vol %) in CB solvent for BHJ solution are shown in Supplementary Table 2. The optimized concentration of DIO was 10 vol% in the SS process, contrary to 3 vol% DIO in the spin coating process.

Supplementary Note 13| Statistics of the performance of SC- and SS-PSCs

The photovoltaic characteristics (V_{oc}, J_{sc}, and *FF*) of the PSCs prove the reliability of the SS-PTB7 based-*n*-PSCs processed in air, as shown in Supplementary Figure 13.

The photovoltaic characteristics (V_{oc}, J_{sc}, and *FF*) of the PSCs prove the reliability of the SS-PTB7-Th based-*i*-PSCs processed in air, as shown in Supplementary Figure 14.

Supplementary References

1. Reyes-Reyes, M., Kim, K. & Carroll, D. L. High-efficiency photovoltaic devices based on annealed poly(3-hexylthiophene) and 1-(3-methoxycarbonyl)-propyl-1-phenyl-(6,6)C-61 blends. *Appl. Phys. Lett.* **87**, 083506 (2005).
2. Vandewal, K. et al. Varying polymer crystallinity in nanofiber poly(3-alkylthiophene): PCBM solar cells: Influence on charge-transfer state energy and open-circuit voltage. *Appl. Phys. Lett*. **95**, 123303 (2009).
3. Ye, L. *et al*. Remove the residual additives toward enhanced efficiency with higher reproducibility in polymer solar cells, *J. Phys. Chem. C*, **117**, 14920-14928 (2013).
